# The Role of Comorbidities in COVID-19 Severity

**DOI:** 10.3390/v17070957

**Published:** 2025-07-07

**Authors:** Sandra König, Ugne Vaskyte, Maria Boesing, Giorgia Lüthi-Corridori, Joerg Daniel Leuppi

**Affiliations:** 1 University Institute of Internal Medicine, Cantonal Hospital Baselland, 4410 Liestal, Switzerland; sandra.koenig@ksbl.ch (S.K.); ugnevaskytee@gmail.com (U.V.); giorgia.luethi-corridori@ksbl.ch (G.L.-C.); 2 Faculty of Medicine, University of Basel, 4056 Basel, Switzerland; 3 PPCR Program, Harvard T. H. Chan School of Public Health, Boston, MA 02115, USA

**Keywords:** SARS-CoV-2, disease severity, comorbidities

## Abstract

Background: COVID-19 has led to significant global morbidity and mortality, with clinical outcomes varying widely among individuals. Understanding the impact of comorbidities on COVID-19 outcomes is essential for improving patient management. To date, analyses of comorbidities affecting COVID-19 severity in a heterogeneous Swiss cohort across multiple outbreak waves are unavailable. The objective of this study was to explore the role of comorbidities on COVID-19 severity in hospitalized patients from a diverse Swiss cohort and to evaluate the association between comorbidities and specific in-hospital complications. Methods: This retrospective, observational, single-center study included adult patients who were hospitalized for COVID-19 for at least one night at the Cantonal Hospital Baselland, Switzerland (KSBL), between March 2020 and December 2021. Logistic regression analyses adjusted for age and gender were performed to analyze the association between comorbidities and critical condition (defined as severe disease or in-hospital death) and complications. Results: A total of 1124 patients were included in the study (median age 66, range 19–100 years, 60% male). A total of 76% of patients had at least one comorbidity. The most common comorbidities were arterial hypertension (47%), obesity (27%), and diabetes mellitus (24%). Overall, 16% of patients experienced a critical condition, and 25.5% had any type of complication. Patients without comorbidities had the lowest rates of critical condition (5.3%) and complications (10.2%). Obesity (OR 2.01, *p* < 0.001), diabetes mellitus (OR 1.67, *p* = 0.004), arterial hypertension (OR 1.65, *p* = 0.006), arrhythmia (OR1.87, *p* = 0.003), and chronic obstructive pulmonary disease (OR 2.72, *p* < 0.001) were found to be associated with critical condition. The most frequently observed complication was acute kidney failure, affecting 17.1% of the study population, while patients with arrhythmia showed the highest overall complication rate (42%). Conclusions: Our findings are consistent with previous research, confirming the relevance of specific comorbidities as key risk factors for critical COVID-19 outcomes. Among all comorbid conditions evaluated, asthma appeared to have the least impact on disease severity. Future research should focus on the impact of the combination of comorbidities on the disease severity of COVID-19, as well as the long-term effects of COVID-19 for patients with certain comorbidities.

## 1. Introduction

The COVID-19 pandemic caused by the Severe Acute Respiratory Virus 2 (SARS-CoV-2) was a major public health challenge with profound epidemiological and clinical implications [1]. It remains a highly relevant issue; in fact, the current global seroprevalence of COVID-19 is approximately 59.2%, and the infection fatality rate ranges between 0.5% and 1% [2,3]. Between March 2020 and December 2021, five distinct waves of COVID-19 were officially documented, characterized by substantial variability in infection rates, hospitalizations, and clinical severity [4]. COVID-19 manifests with a wide spectrum of clinical presentations and outcomes. Most people have mild to moderate symptoms and recover within a few days, whereas some people progress to a severe condition and require hospitalization [5]. Certain individuals are at particularly high risk of severe COVID-19 [5]. Therefore, defining risk groups is crucial, as it forms the basis for public health recommendations and control measures, such as vaccination plans [6]. In order to define risk groups, identifying potential risk factors is crucial. This enables the early recognition of patients with a poor prognosis, ultimately helping to reduce mortality rates.

Considerable research on risk factors for severe illness has already been conducted globally since the outbreak of the pandemic in early 2020 [7]. In China, for example, studies have highlighted male sex, advanced age, and pre-existing comorbidities as key contributors to increased morbidity of COVID-19 [8]. Similarly, research from India showed that increased COVID-19-related morbidity of older individuals is primarily attributable to age-associated comorbidities prevalent within this demographic [9]. In general, patients with pre-existing comorbidities have worse disease progression compared to those without comorbidities [10]. Similar findings were observed in Switzerland. A study conducted in 17 Swiss hospitals (n = 3590) between February and August 2020 showed that increasing age is the most important risk factor for in-hospital mortality among hospitalized COVID-19 patients, along with male sex, followed by the presence of comorbidities such as renal diseases, chronic respiratory or cardiovascular diseases, oncological malignancies, and dementia [6]. Another study conducted during the first wave of the pandemic across 20 Swiss hospitals (n = 3645) reported hypertension (61.7%, n = 1481), cardiovascular diseases (39.5%, n = 948), and diabetes mellitus (27.5%, n = 660) as the most common pre-existing conditions among hospitalized patients [11]. These Swiss studies were conducted during the early phase of the pandemic, a period when the majority of individuals lacked immunological preparedness against the virus, and no vaccines were yet available. Nonetheless, the results underline the crucial role that comorbidities play in the disease severity of COVID-19.

In the context of disease severity, it is important to recognize that COVID-19 can cause a wide range of extra-pulmonary complications [12]. Therefore, while pulmonary complications are a primary concern, healthcare professionals should also consider the potential effects of COVID-19 on other organs, with—as research has shown—particular attention to the kidneys [13]. Consequently, investigating the associations between specific comorbidities and their related complications is both necessary and valuable for improving patient management. Studies have, for example, linked obesity to an increased risk of complications associated with COVID-19 [14,15].

A comprehensive understanding of the interplay between comorbidities and COVID-19 complications is essential for identifying high-risk populations. This knowledge not only informs clinical management but also reinforces the importance of targeted preventive measures, such as vaccination, in mitigating severe outcomes. To date, analyses investigating the impact of comorbidities on COVID-19 severity in a heterogeneous Swiss cohort, including subsequent outbreak waves beyond the first, are unavailable. Additionally, there is a lack of analyses investigating the relationship between comorbidities and extra-pulmonary complications within this patient group and timeframe. The primary objective of this study was to examine the association between various comorbidities—obesity, diabetes mellitus, arterial hypertension, heart failure, coronary heart, arrhythmia, active cancer, chronic obstructive pulmonary disease (COPD), asthma bronchiale, rheumatologic diseases, and chronic kidney disease—and the severity of COVID-19 in hospitalized patients from a diverse Swiss group in 2020 and 2021. Since age and gender are known to be essential risk factors, parameters will be analyzed with respect to these potential confounders. Secondary objectives included evaluating the association between comorbidities and specific in-hospital complications. The results may help identify high-risk patients within this population, which can guide prevention strategies and optimize clinical management for those at greater risk.

## 2. Materials and Methods

### 2.1. Study Design and Setting

This study was a retrospective, observational, single-center study. Adult patients who were hospitalized for COVID-19 for at least one night at the Cantonal Hospital Baselland, Switzerland (KSBL), a public teaching hospital serving a population of about 290,000, between March 2020 and December 2021 and who fulfilled the eligibility criteria were included in this study.

### 2.2. Study Population

Adult patients (18 years or older) hospitalized with laboratory-confirmed COVID-19 as their primary diagnosis were eligible for inclusion in this study. Patients who declined general consent for the use of health-related data and biological samples for research purposes were excluded from the study. Additionally, patients for whom COVID-19 was not the primary diagnosis were excluded. If there were multiple eligible cases of one patient during the study period, only the first was included in order to avoid bias.

### 2.3. Outcomes

The primary outcome of the study was the severity of COVID-19 disease, defined based on the 10-point WHO progression scale [16], in an adapted version for hospitalized patients as presented in Table 1.

Disease severity was further grouped into a binary classification, defining “critical condition” as the composite of severe disease or in-hospital death for the use in logistic regression analyses (see Section 2.5). This composite outcome was chosen because the two are closely related and cannot be strictly separated in terms of severity. Admission to intensive care, which is inevitable for advanced procedures such as mechanical ventilation, vasoactive drugs, and additional organ support, depends on many individual factors, personal preferences, and beliefs [17]. Since these procedures can potentially prevent in-hospital death, severe illness and in-hospital death need to be considered together.

Secondary exploratory outcomes included the following in-hospital complications:

– Ventilator-associated pneumonia (VAP);
– Acute kidney failure (AKF);
– Multi-organ failure (MOF);
– Thromboembolic complications, including:
  - Pulmonary embolism
  - Deep vein thrombosis
  - Acute arterial occlusion
  - Cerebrovascular insult
  - Amputation
  - Disseminated intravascular coagulation;
– Cardiac complications, including:
  - Heart failure
  - Acute coronary syndrome
  - Cardiac arrest;
– Neurological complications, including:
  - Delirium
  - Encephalopathy
  - Meningoencephalitis
  - Neuromuscular disease
  - Polyneuropathy.

The inclusion of specific complications as secondary outcomes allows for a more nuanced understanding of how comorbidities contribute to the clinical disease outcome.

### 2.4. Data Collection and Management

A list of inpatients coded with the ICD-10 code for COVID-19 (U07.1 COVID-19, virus identified) as their primary diagnosis between March 2020 and December 2021 was extracted from the institution’s control system. After verification of eligibility according to the criteria listed in 4.2, relevant additional patient data were manually extracted from the electronic health records. Data were entered into a REDCap^®^ (Research Electronic Data Capture) database.

### 2.5. Statistical Analysis

Patient data were analyzed descriptively and presented as absolute and relative frequencies or median and interquartile ranges (IQRs). To further assess the association between comorbidities and outcomes, we performed logistic regression analyses adjusted for age and gender as follows. For each of the comorbidities—obesity, diabetes mellitus, arterial hypertension, heart failure, coronary heart disease, arrhythmia, malignant disease, COPD, asthma bronchiale, and rheumatologic disease—we performed one logistic regression with the outcome of interest as the dependent variable, and the specific comorbidity, age, and gender as independent variables. The results were reported as odds ratios (ORs) with 95% confidence intervals (CIs). A *p*-value <0.05 was considered statistically significant.

In order to assess the interplay between different comorbidities and their effect on the reported outcomes, we additionally performed multivariable logistic regression analyses for the outcomes “critical condition” and “complications”, with the outcome of interest as the dependent variable and all comorbidities, plus age and gender, as independent variables. The corresponding results are reported in Appendix A. Analyses were performed with REDCap^®^, v. 14.7.5, and with R version 4.1.0.

## 3. Results

### 3.1. Patient Characteristics

**Figure 1:**
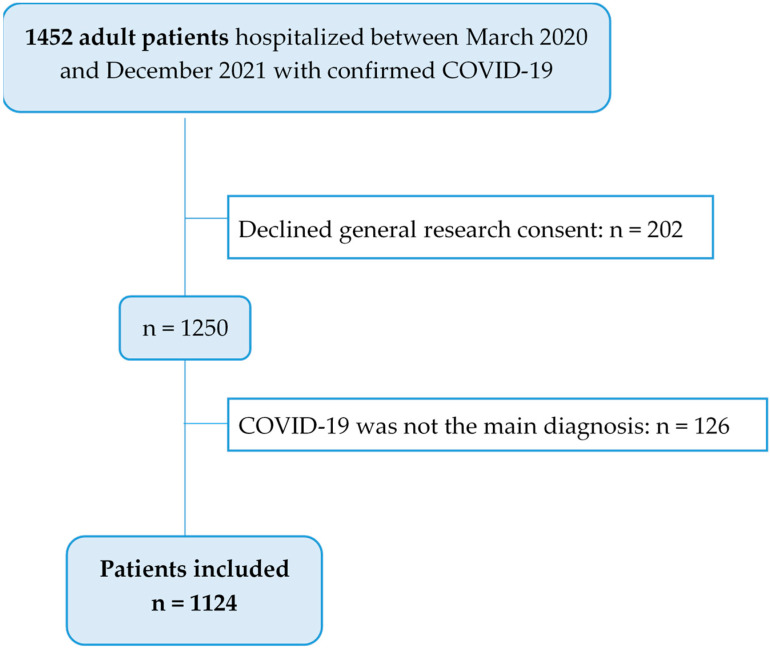
The Flow Chart included patients.

Between March 2020 and December 2021, 1452 patients were hospitalized with confirmed COVID-19 at the KSBL. Following the exclusion of 202 patients who withheld consent for the use of their data for research purposes and 126 who were excluded because COVID-19 was not the main diagnosis. A total of 1124 patients were included in this study. A flowchart of patient inclusion and exclusion is presented in Figure 1.

Table 2 summarizes the characteristics of the included patients. The median age was 66 years, ranging from 19 to 100 (IQR: 54–79), and 59.5% of the patients were male (n = 669).

Three-quarters of the patients had at least one comorbidity (n = 850). The most prevalent comorbidity was arterial hypertension (46.7%, n = 525), followed by obesity (27.0%, n = 303) and diabetes mellitus (23.8%, n = 268). A total of 84 individuals (7.5%) presented with all three of these comorbidities. Chronic heart conditions—namely heart failure, coronary heart disease, or arrhythmia—were reported in a total of 284 patients (25.3%). An obstructive respiratory condition was present in 124 patients (11.0%), with COPD in 5.5% (n = 62) and asthma bronchiale in 5.7% (n = 64). Two patients were suffering from both asthma and COPD. The majority of the included patients were hospitalized at a stage of the pandemic when COVID-19 vaccination was not yet available for their specific risk group (58.4%, n = 656). Out of 293 patients for whom vaccination was available, 108 (36.9%) were vaccinated.

### 3.2. Primary Outcome: Comorbidities and Disease Severity

Overall, the vast majority of patients (83.9%, n = 943) had moderate disease, while 64 patients survived a severe illness (5.7%), and in-hospital mortality was 10.4% (n = 117). When stratifying patients by comorbidities, moderate disease severity was still the most prevalent category across all groups, with a range from 61.3 to 93.8%. Out of the 274 patients without any reported comorbidities, 260 (94.9%) had a moderate disease progression, 12 (4.4%) survived a severe illness, and 2 patients (0.7%) died during hospitalization. The highest in-hospital mortality was observed in the groups of patients with heart failure (30.8%, n = 8), COPD (29%, n = 18), and active cancer (27.6%, n = 16). The highest rate of severe condition (severe disease or in-hospital death) was observed in patients with COPD (38.7%, n = 24), followed by heart failure (30.8%, n = 8) and arrhythmia (30.7%, n = 47). Notably, within the total of 64 individuals diagnosed with asthma bronchiale, no fatalities were observed. Disease progression per comorbidity group is reported in Table 3.

**Figure 2:**
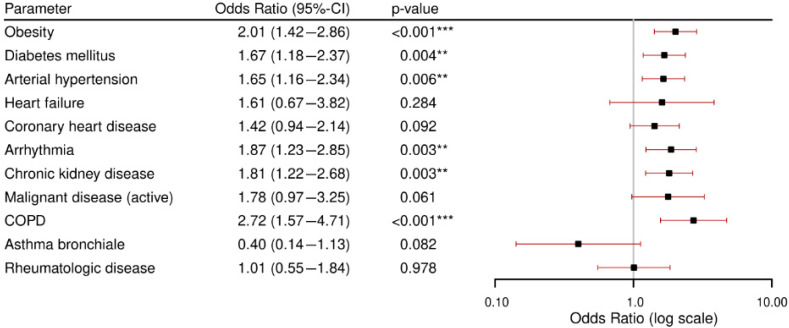
Results of the logistic analyses for critical conditions per comorbidity. All models were adjusted for age and gender. CI: confidence interval. COPD: chronic obstructive pulmonary disease. ** = *p*-value < 0.05, *** = *p*-value < 0.001.

Figure 2 displays the results of the logistic regression analyses for the binary composite outcome “critical condition” for each comorbidity, adjusted for age and gender. The highest odds for critical condition were found in patients with COPD (OR 2.72, *p* < 0.001) and obesity (OR 2.01, *p* < 0.001), followed by arrhythmia (OR 1.87 [1.23–2.85], *p* = 0.003), diabetes mellitus (OR 1.67 [1.18–2.37], *p* = 0.004), and arterial hypertension (OR 1.65 [1.16–2.34], *p* = 0.006). The other comorbidities had no significant associations with critical conditions.

### 3.3. Secondary Outcome: Comorbidities and Complications.

Out of a total of 1124 patients, 287 patients (25.5%) suffered from at least one of the complications under investigation: VAP, AKF, MOF, thromboembolic complications, cardiac complications, and neurological complications. In patients with no comorbidities (n = 274), complications were less frequent, only occurring in 10.2% (n = 28).

Overall, the most reported complication was AKF (17.1%, n = 192), followed by thromboembolic complications (5.5%, n = 62) and cardiac complications (4.8%, n = 54). The highest overall complication rate was observed in patients with chronic kidney disease (48.0%, n = 108/225), followed by patients with arrhythmia (41.8%, n = 64/153) and patients with arterial hypertension (36.6%, n = 192/525).

In patients with comorbidities associated with metabolic syndrome, AKF was by far the most frequently reported complication: 20.5% (n = 62) in patients with obesity, 22% (n = 59) in patients with diabetes mellitus, and 24.6% (n = 129) in patients with arterial hypertension. On the other hand, MOF was observed only in rare cases within this patient population: in 6 out of 303 (2%) patients with obesity, 5 out of 268 (1.9%) patients with diabetes mellitus, and 8 out of 525 (1.5%) patients with arterial hypertension. The most frequently reported complication type among individuals with heart failure was cardiac complications at 30.8% (n = 8), followed by AKF at 23.1% (n = 6), whereas no cases of thromboembolic complications were reported within this patient group. Within patients with chronic respiratory conditions, AKF was the most frequently reported complication: 24.2% (n = 15) in patients with COPD and 18.8% (n = 12) in patients with asthma bronchiale. Detailed complication rates are reported in Table 4.

**Figure 3:**
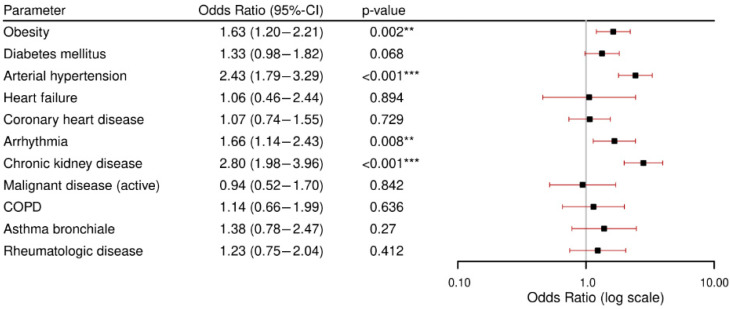
Results of the logistic analyses for the occurrence of complications per comorbidity. All models were adjusted for age and gender. CI: confidence interval. COPD: chronic obstructive pulmonary disease. ** = *p*-value < 0.05, *** = *p*-value < 0.001.

Regression analyses adjusted for age and gender revealed that obesity, arterial hypertension, arrhythmia, and chronic kidney disease were associated with the occurrence of in-hospital complications (details see Figure 3). The highest odds were found in patients with chronic kidney disease (OR 2.9, *p* < 0.001) and arterial hypertension (OR 2.4, *p* < 0.001), followed by arrhythmia (OR 1.66, *p* = 0.008) and obesity (OR 1.63, *p* = 0.002). None of the other comorbidities was associated with the occurrence of in-hospital complications.

## 4. Discussion

This retrospective cohort study evaluated the impact of certain comorbidities on the severity of COVID-19 disease. Additionally, the impact of comorbidities on COVID-19-related complications was outlined. Our study has four main findings:

1. Patients with comorbidities had a 1.4-fold rate of severe disease outcomes and a 20-fold in-hospital mortality rate compared to individuals without comorbidities, emphasizing the impact of pre-existing conditions. Moderate disease was the most frequent outcome across all observed sub-groups.
2. Obesity, diabetes, and arterial hypertension were the most prevalent comorbidities and were significantly associated with a critical condition (severe disease or in-hospital death) after adjusting for age and gender. It should be noted that while comorbidities such as CKD and arrhythmia showed higher odds ratios, their lower prevalence means that their overall population-level impact differs from more common conditions like hypertension or obesity. Our focus in this section is, therefore, on conditions that combine both high prevalence and a significant risk increase. From a public health perspective, this dual perspective is critical for resource planning and preventive strategies.
3. Patients with chronic kidney disease and arterial hypertension were at particularly high risk for developing in-hospital complications. Across all analyzed comorbidities, acute kidney failure was the most frequently observed complication.
  Obstructive respiratory conditions (COPD and asthma bronchiale) were relatively uncommon. Notably, asthma bronchiale appeared to have a potentially protective effect against severe outcomes.

The demographic characteristics of our study population, with a median age of 66 years and a male predominance, align with other studies conducted in Switzerland [11,18,19].

### 4.1. The Role of Comorbidities in Disease Severity and Complications

#### 4.1.1. Disease Severity Across All Observed Comorbidities

Irrespective of comorbidities, 83.9% of the individuals in our study population experienced a moderate disease course, requiring hospitalization but no form of advanced respiratory support (NIV, HFNO, or intubation). This suggests that most cases were not severe, even in patients with pre-existing conditions. However, many patients may have declined ICU treatment despite needing advanced respiratory support, which changes the interpretation of this finding. Reasons for declining treatment in the ICU may be old age, serious underlying illnesses, cognitive impairments, or living in institutions [20]. The expected quality of life after ICU and survival chances also play a key role, as patients are more likely to decline intensive care if the prognosis is poor [21]. Consequently, some severe cases may be underrepresented in our dataset because patients who refused ICU admission were not classified as ‘critical’ despite having a potentially severe clinical condition that would have otherwise qualified for ICU-level care. Although the proportion of these patients was likely small, this should be taken into account. 

#### 4.1.2. Comorbidities in Metabolic Syndrome

The most prevalent comorbidity observed in our study was arterial hypertension (46.7%). Other studies underpin this finding, in which arterial hypertension is one of the most prevalent comorbidities among COVID-19 patients and is even associated with increased risk of ICU admission and mortality [6,22,23]. Obesity was the second most observed comorbidity in our study (27.0%). Diabetes mellitus, which occurred in 23.8% of our study population, is verifiably another common illness that significantly increases the risk of severe COVID-19 and mortality [6,22,23]. Prior research suggests that metabolic syndrome is associated with increased COVID-19 severity and mortality [24,25,26].

**Figure A1:**
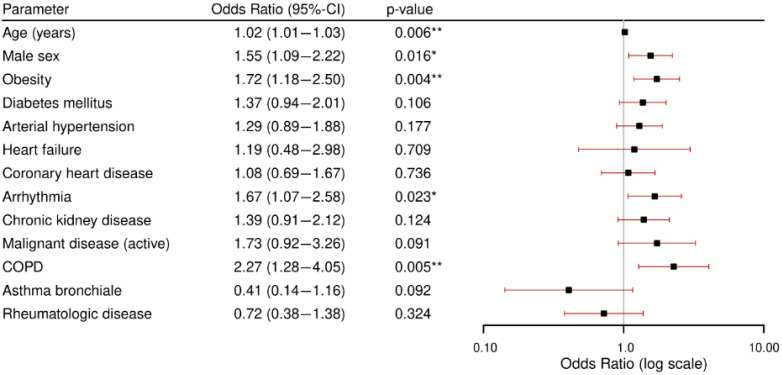
Forest plot full multivariable model for critical condition. * = *p*-value < 0.05, ** = *p*-value < 0.01.

Also, after adjusting for age and gender, these comorbidities demonstrate a significantly higher risk of severe disease outcomes in our study population. However, when adjusted for all other comorbidities, only obesity among the three remains with a statistically increased independent risk for critical condition (OR 1.72 [1.18–2.50], *p* = 0.004, see Figure A1 in Appendix A). This can be explained by the fact that diabetes mellitus as well as arterial hypertension often coexist with other comorbidities, which themselves contribute to worse COVID-19 outcomes. The loss of statistical significance suggests that the increased risk is more likely due to these other comorbidities rather than diabetes mellitus or arterial hypertension alone.

Nonetheless, it has to be noticed that only 12.2% of the patients with arterial hypertension, 13% of the patients with obesity, and 15.8% of patients with diabetes mellitus in our study population were vaccinated (for more detailed information, see Table A1 in Appendix A). Given that COVID-19 vaccination only became available in Switzerland in December 2020, near the midpoint of our study period, vaccination was not widely present in this cohort and is unlikely to have significantly influenced our findings.

After correction for age and gender, patients with obesity and arterial hypertension show an overall significantly increased risk for complications. Within patients with arterial hypertension, obesity, and/or diabetes mellitus, acute kidney failure was by far the most frequently reported complication. This can be explained by the fact that a combination of chronic inflammation, oxidative stress, elevated uric acid levels, and glomerular hyperfiltration in patients with metabolic syndrome contributes to the deterioration of renal function and enhances susceptibility to acute kidney failure [27,28,29,30].

#### 4.1.3. Cardiovascular Comorbidities

A meta-analysis by Sabatino et al. outlines that cardiovascular comorbidities are significantly associated with increased mortality and more severe outcomes in COVID-19 patients [31]. These findings are supported by another study showing that patients with cardiovascular disease have higher mortality and more severe clinical outcomes [32].

**Figure A2:**
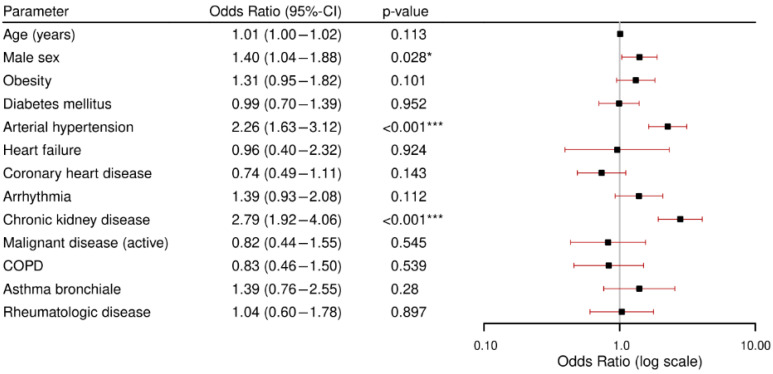
Forest plot full multivariable model for complications. * = *p*-value < 0.05, *** = *p*-value < 0.001.

Interestingly, in our study, only two of the four analyzed cardiovascular conditions were significantly associated with a critical disease course after adjusting for age and gender: arterial hypertension (OR 1.65 [1.16–2.34], *p* = 0.006) and arrhythmia (OR 1.87 [1.23–2.85], *p* = 0.003). However, after adjusting for all comorbidities, including age and gender, only arrhythmia remained statistically significant (OR 1.72 [1.11–2.66], *p* = 0.015) (see Figure A1 in Appendix A). In terms of complications, the univariate as well as the multivariable analysis showed a significant association with a critical disease outcome for arterial hypertension (OR 2.43 [1.79–3.29], *p* < 0.001 versus OR 2.23 [1.62–3.06], *p* < 0.001) and arrhythmia (OR 1.66 [1.14–2.43], *p* = 0.008 versus OR 1.54 [1.04–2.27], *p* = 0.0.31) (see Figure A2 in Appendix A).

#### 4.1.4. Chronic Kidney Disease

Patients with CKD in our cohort demonstrated significantly worse clinical outcomes, including higher rates of critical condition and also higher risk of complications. These findings are in line with previous studies identifying CKD as an independent risk factor for critical COVID-19 progression and poor prognosis, even after adjusting for age and other comorbidities [33]. Several pathophysiological mechanisms may contribute to this heightened vulnerability, including impaired immune responses, chronic systemic inflammation, and a pro-thrombotic state associated with renal impairment. In our cohort, only a small number of patients suffered from end-stage kidney disease, and their in-hospital mortality rate (22.2%, 2 out of 9) was not higher than that of patients with lower-stage CKD (25.9%, 56 out of 216).

Our data align with the results of a systematic review and meta-analysis by Chung et al., which reported that individuals with CKD and COVID-19 had a markedly higher incidence of death compared to those without CKD, with an incidence rate ratio of 10.26 [34]. Similarly, Ozturk et al. found significantly elevated ICU admission (39.4%) and in-hospital mortality rates (28.4%) among patients with CKD [35]. Flythe et al. further supported this evidence, showing that non-dialysis-dependent CKD patients admitted to the ICU had a higher risk of 28-day in-hospital death (adjusted hazard ratio 1.25) [36].

#### 4.1.5. Obstructive Respiratory Comorbidities

In our study, COPD was significantly associated with critical outcomes, with an OR of 2.72 [1.57–4.71], *p* < 0.001. Also, when adjusted for all comorbidities in a multivariable analysis, the results remain significant (OR 2.35 [1.32–4.16], *p* = 0.003) (see Figure A1 in Appendix A). These findings are not surprising, as studies show that COPD is a significant risk factor for critical conditions and poorer prognoses in COVID-19 patients [37,38]. Interestingly, COPD was not significantly associated with an increased risk for in-hospital complications when corrected for age, gender, and comorbidities under investigation (OR of 0.94 [0.53–1.67], *p* = 0.832) (see Figure A2 in Appendix A).

In contrast, asthma bronchiale appeared to have a potentially protective effect, with only four (6.2%) of affected patients experiencing a critical condition, and none of them dying in hospital. Accordingly, the logistic regression revealed an OR of 0.4. However, the wide 95% confidence interval (0.14–1.13) and the lack of statistical significance (*p* = 0.082) warrant a cautious interpretation of this finding. When corrected for all comorbidities in a multivariable analysis, results remain similar (see Figure A1 in Appendix A). Nevertheless, existing evidence indicates that asthma bronchiale may indeed confer a protective effect in patients with COVID-19. A meta-analysis of 15 cohort studies found that COVID-19 patients with asthma have a significantly reduced risk of mortality (pooled HR = 0.88 [0.82–0.95]), and a pooled analysis of 57 studies showed a 13% lower risk of hospitalization compared to non-asthmatics [39,40]. Evidence suggests that type 2 airway inflammation, common in asthma, may lower susceptibility to COVID-19 due to reduced expression of ACE2 and TMPRSS2 receptors, with corticosteroids further decreasing their expression, potentially providing additional protection [41].

When interpreting this comparison of obstructive respiratory conditions, it is noteworthy that only 14.5% of patients with asthma bronchiale, compared to 18.9% of those with COPD, were vaccinated at the time of hospitalization (see Table A1 in Appendix A).

### 4.2. Strengths, Limitations, and Further Research

This study covers a critical period of the COVID-19 pandemic, from the initial outbreak through four subsequent waves, and provides valuable insights into the role of comorbidities within a highly heterogeneous Swiss cohort. By analyzing a diverse patient population, it highlights the independent impact of pre-existing conditions on disease severity and outcomes, contributing to a better understanding of risk factors in different patient groups.

This study has several limitations. Its single-center, retrospective design may limit generalizability, and data quality depends on accurate documentation, leaving possible gaps. Due to small sample sizes, stratification by virus variants or treatment was not performed, but it could improve analysis in a larger cohort. Additionally, viral genotyping data were not available at the individual patient level, as this was not part of routine clinical practice during the study period. As a result, our analysis does not account for potential differences in outcomes related to specific SARS-CoV-2 variants.

Additionally, incomplete documentation of dyslipidaemia restricted insights into the role of lipid abnormalities in disease outcomes. Another limitation is the absence of a systematically recorded solid organ transplant status. While the literature demonstrates that transplant recipients are at higher risk for severe COVID-19 outcomes, this comorbidity was not explicitly coded in our dataset, potentially leading to an underrepresentation of this high-risk group in our analysis.

Furthermore, our dataset was limited to cases from the initial two years of the pandemic, with data collection concluding in December 2021. While this restricts our ability to compare outcomes with later waves and newer viral variants, our findings remain relevant, particularly for patients with comorbidities who continue to face elevated risks. Moreover, all data were manually verified from clinical records to ensure accuracy, as comorbidities are often incompletely or inaccurately documented in structured hospital systems where coding is primarily reimbursement-driven. This intensive process requires substantial resources and makes the retrospective extension of the dataset currently infeasible. Future analyses, including post-2022 data, would be valuable to contextualize these early patterns in light of evolving clinical and public health landscapes.

Another limitation is the lack of systematically documented vaccination data. Since COVID-19 vaccination began in Switzerland in December 2020 and was not yet widely available for much of the study period, vaccination status could not be reliably included in our analyses. This absence limits the interpretation of outcomes within the context of evolving immunization strategies. Future studies should incorporate vaccination data, particularly for high-risk groups, to assess its modifying effect on disease severity.

Finally, the long-term effects of the disease under considering comorbidities, were not analyzed in this study.

Future research should focus on the combined impact of comorbidities on disease severity and the long-term outcomes of COVID-19, especially as new viral variants and treatment paradigms continue to evolve.

Although our study focuses on data from the early pandemic period, its findings remain clinically relevant in the current landscape. Individuals with pre-existing conditions continue to face elevated risks, even as newer SARS-CoV-2 variants have emerged with different transmission and severity profiles. Recent literature suggests that the association between comorbidities and poor outcomes persists despite vaccination efforts and therapeutic advancements [42]. Therefore, our results may serve as a foundation for understanding vulnerability patterns that still impact patient outcomes today, including the risk of long COVID and complications related to newer strains.

## 5. Conclusions

The findings of this study highlight the substantial influence of specific comorbidities on COVID-19 severity and in-hospital mortality within a diverse Swiss cohort between March 2020 and December 2021—arguably the most dynamic phase of the COVID-19 pandemic.

By analyzing a broad range of common in-hospital complications, this study contributes to a deeper understanding of COVID-19 and may support early patient management by identifying key risk factors. By performing different univariate and multivariable analyses as well as considering vaccination status, confounding effects like age, gender, or the influence of other illnesses could be identified and involved in the interpretation of the results.

Comorbidities commonly associated with metabolic syndrome– namely, obesity, diabetes, and arterial hypertension—appear to increase the risk of critical outcomes, even when adjusted for age and gender. Notably, CKD was identified as one of the most impactful comorbidities in terms of both critical course and complication risk. Among cardiovascular comorbidities, arterial hypertension and arrhythmia were significantly associated with critical disease progression, even after adjusting for age and gender. However, when all analyzed cardiovascular conditions were corrected for age, gender, as well as for other comorbidities, only arrhythmia remained significantly associated with critical outcome. Cardiac diseases, active cancer, and COPD showed a higher mortality rate compared to other comorbidities. Asthma had the least impact on COVID-19 severity among all comorbidities. Other studies have confirmed a possible protective effect of asthma.

Overall, our study aligns with the findings of similar research, reinforcing the role of certain comorbidities as potential risk factors. It demonstrates that these risk factors were relevant not only at the onset of the pandemic but also throughout subsequent waves, encompassing a wide range of vaccination statuses, virus variants, and varying levels of immunologic preparedness among patients. Identifying and managing patients with high-risk profiles remains essential for reducing severe outcomes and optimizing resource allocation in future respiratory pandemics.

## Figures and Tables

**Table 1:** COVID-19 disease progression scale for hospitalized patients, adapted from [16].

Moderate	Hospitalized without oxygen therapy Hospitalized with oxygen by a nasal prong or mask
Severe	Hospitalized with oxygen by NIV or HFNO Hospitalized and intubated, incl. patients with additional organ support (dialysis, ECMO, vasoactive drugs)
In-hospital death	

NIV = noninvasive ventilation, HFNO = high-flow nasal oxygen, ECMO = extracorporeal membrane oxygenation.

**Table 2:** Patient characteristics.

	Overall n = 1124 (100.00%)	Missing Data
Demographics		
Age in years, median [IQR] (range)	66 [54–79] (19–100)	
Male, n (%)	669 (59.5)	
Female, n (%)	455 (40.5)	
BMI, median [IQR]	27.10 [23.9–30.8]	
Comorbidities		
Obesity, n (%)	303 (27.0)	
Diabetes mellitus, n (%)	268 (23.8)	
Arterial hypertension, n (%)	525 (46.7)	
Heart failure, n (%)	26 (2.3)	
Coronary heart, n (%)	168 (14.9)	
Arrhythmia, n (%)	153 (13.6)	
Chronic kidney disease, n (%)	225 (20)	
End-stage CKD	9 (4)	
Malignant disease (active), n (%)	58 (5.2)	
COPD, n (%)	62 (5.5)	
Asthma bronchiale, n (%)	64 (5.7)	
Rheumatologic disease, n (%)	77 (6.9)	
No comorbidities, n (%)	274 (24.4)	
COVID-19 vaccination status		
Not vaccinated, n (%)	185 (16.5)	n = 175 (15.5%)
Vaccinated, n (%)	108 (9.6)	
No vaccination available, n (%)	656 (58.4)	
Smoking status		
Never, n (%)	127 (11.3)	n = 822 (73.1%)
Former smoker, n (%)	138 (12.3)	
Current smoker, n (%)	37 (3.3)	
Precise diagnosis of COVID-19		
Bilateral pneumonia with SARS-CoV-2, n (%)	783 (69.7)	
Unilateral pneumonia with SARS-CoV-2, n (%)	54 (4.8)	
Upper and lower respiratory tract infection with SARS-CoV, n (%)	169 (15.0)	
ARDS in COVID-19, n (%)	118 (10.5)	

IQR: interquartile range; BMI: body mass index; COPD: chronic obstructive pulmonary disease; ARDS: acute respiratory distress syndrome.

**Table 3:** Comorbidities and disease severity.

	n	Moderate	Severe	Death
Overall	1124	943 (83.9)	64 (5.7)	117 (10.4)
No comorbidity	274	260 (94.9)	12 (4.4)	2 (0.7)
Obesity	303	238 (78.5)	28 (9.2)	37 (12.2)
Diabetes mellitus	268	204 (76.1)	21 (7.8)	43 (16)
Arterial hypertension	525	410 (78.1)	37 (7)	78 (14.9)
Heart failure	26	18 (69.2)	0 (0)	8 (30.8)
Coronary heart	168	124 (73.8)	5 (3)	39 (23.2)
Arrhythmia	153	106 (69.3)	8 (5.2)	39 (25.5)
Chronic kidney disease	225	160 (71.1)	7 (3.1)	58 (25.8)
Active cancer	58	41 (70.7)	1 (1.7)	16 (27.6)
COPD	62	38 (61.3)	6 (9.7)	18 (29)
Asthma bronchiale	64	60 (93.8)	4 (6.2)	0 (0)
Rheumatologic disease	77	62 (80.5)	4 (5.2)	11 (14.3)

COPD: chronic obstructive pulmonary disease.

**Table 4:** Comorbidities and complications.

	n	Any	VAP	AKF	MOF	Thromb	Cardio	Neuro
Overall	1124	287 (25.5)	49 (4.4)	192 (17.1)	13 (1.2)	62 (5.5)	54 (4.8)	47 (4.2)
No comorbidity	274	28 (10.2)	6 (2.2)	15 (5.5)	1 (0.4)	10 (3.6)	2 (0.7)	3 (1.1)
Obesity	303	92 (30.4)	22 (7.3)	62 (20.5)	6 (2)	14 (4.6)	19 (6.3)	17 (5.6)
Diabetes mellitus	268	87 (32.5)	17 (6.3)	59 (22)	5 (1.9)	14 (5.2)	17 (6.3)	12 (4.5)
Arterial hypertension	525	192 (36.6)	34 (6.5)	129 (24.6)	8 (1.5)	37 (7)	42 (8)	32 (6.1)
Heart failure	26	9 (34.6)	0 (0)	6 (23.1)	1 (3.8)	0 (0)	8 (30.8)	1 (3.8)
Coronary heart	168	56 (33.3)	5 (3)	38 (22.6)	3 (1.8)	4 (2.4)	20 (11.9)	5 (3)
Arrhythmia	153	64 (41.8)	5 (3.3)	43 (28.1)	4 (2.6)	12 (7.8)	25 (16.3)	8 (5.2)
Chronic kidney disease	225	108 (48)	6 (2.7)	93 (41.3)	6 (2.7)	16 (7.1)	28 (12.4)	9 (4)
Active cancer	58	17 (29.3)	1 (1.7)	13 (22.4)	0 (0)	3 (5.2)	1 (1.7)	1 (1.7)
COPD	62	21 (33.9)	5 (8.1)	15 (24.2)	2 (3.2)	3 (4.8)	7 (11.3)	5 (8.1)
Asthma bronchiale	64	18 (28.1)	4 (6.2)	12 (18.8)	0 (0)	4 (6.2)	0 (0)	4 (6.2)
Rheumatologic disease	77	26 (33.8)	2 (2.6)	19 (24.7)	2 (2.6)	2 (2.6)	7 (9.1)	4 (5.2)

COPD: chronic obstructive pulmonary disease. VAP: ventilator-associated pneumonia. AKF: acute kidney failure. MOF: multi-organ failure. Thromb: thromboembolic complication. Cardio: Cardiac complication. Neuro: neurological complication.

## Data Availability

The data presented in this study are available upon reasonable request from the corresponding author. The data are not publicly available due to restrictions on data privacy.

## Appendix A

**Table A1:** Vaccination status per comorbidity.

	n	Vacc_Not_Available	Vacc_Yes	Vacc_No	Missing
Overall	1124	656 (69.1)	108 (11.4)	185 (19.5)	175
No comorbidity	274	145 (62.5)	16 (6.9)	71 (30.6)	42
Obesity	303	187 (71.4)	34 (13)	41 (15.6)	41
Diabetes mellitus	268	155 (69.8)	35 (15.8)	32 (14.4)	46
Arterial hypertension	525	319 (72.3)	54 (12.2)	68 (15.4)	84
Heart failure	26	16 (69.6)	4 (17.4)	3 (13)	3
Coronary heart	168	102 (75)	19 (14)	15 (11)	32
Arrhytmia	153	84 (65.1)	21 (16.3)	24 (18.6)	24
Active cancer	58	33 (64.7)	10 (19.6)	8 (15.7)	7
COPD	62	39 (73.6)	10 (18.9)	4 (7.5)	9
Asthma bronchiale	64	45 (81.8)	8 (14.5)	2 (3.6)	9
Rheumatologic disease	77	50 (73.5)	13 (19.1)	5 (7.4)	9
Chronic kidney disease	225	122 (67.8)	37 (20.6)	21 (11.7)	45
